# Combining Zeolites with Early-Maturing Annual Legume Cover Crops in Rainfed Orchards: Effects on Yield, Fatty Acid Composition and Polyphenolic Profile of Olives and Olive Oil

**DOI:** 10.3390/molecules28062545

**Published:** 2023-03-10

**Authors:** Sandra Martins, Ermelinda Silva, Cátia Brito, Luís Pinto, Carlos Martins-Gomes, Alexandre Gonçalves, Margarida Arrobas, Manuel Ângelo Rodrigues, Carlos M. Correia, Fernando M. Nunes

**Affiliations:** 1 CITAB—Centre for the Research and Technology of Agro-Environmental and Biological Sciences, University of Trás-os-Montes and Alto Douro, 5000-801 Vila Real, Portugal; 2 MORE—Collaborative Laboratory Mountains of Research, Brigantia Ecopark, 5300-358 Bragança, Portugal; 3 Inov4Agro—Institute for Innovation, Capacity Building and Sustainability of Agri-Food Production, University of Trás-os-Montes and Alto Douro, 5000-801 Vila Real, Portugal; 4 CQ-VR—Food and Wine Chemistry Laboratory, Chemistry Research Centre—Vila Real, University of Trás-os-Montes e Alto Douro, 5000-801 Vila Real, Portugal; 5 CIMO—Centro de Investigação de Montanha, Instituto Politécnico de Bragança, 5300-253 Bragança, Portugal

**Keywords:** leguminous cover crops, olive tree, olive oil quality, soil tillage, zeolites

## Abstract

Under climate change threats, there is a growing need to adapt the conventional agronomic practices used in rainfed olive orchards by sustainable practices, in order to ensure adequate crop yield and olive oil quality and to preserve soil health. Therefore, for two years, the effects of conventional tillage practice (T) and two sustainable soil management strategies, a leguminous cover crop (LC) and its combination with natural zeolites (ZL), on the yield, fatty acid composition, polyphenolic profile and quality indices of olive fruits and oil were evaluated. Crop yield was significantly increased by LC and ZL in the first year. Although in the second year no significant differences were verified, the cumulative yield increased significantly by 31.6% and 35.5% in LC and ZL trees, respectively. LC enhanced the moisture and size of olives, while ZL increased, in general, the concentrations of oleuropein, verbascoside, caffeic acid and epicatechin, as well the oleic/linoleic ratio in fruits and the levels of 3,4-dihydroxyphenylglycol, tyrosol, verbascoside and caffeic acid in olive oil. Despite the higher concentration of total phenols in the fruits and oil from T trees in the warmer and dryer year, the quality of the oil decreased, mainly when compared with ZL, as evidenced by the peroxide value and K232 and K270 coefficients. In short, both sustainable soil management strategies appear to be promising practices to implement in olive orchards under rainfed conditions, but the innovative strategy of combining zeolites with legume cover crops, first reported in the present study, confers advantages from a nutritional and technological point of view. Nevertheless, studies subjected to the long-term use of these practices should be conducted to ensure the sustainability of the crop yield and olive oil quality.

## 1. Introduction

The olive tree (*Olea europaea* L.) is one of the most cultivated crops in the Mediterranean region, where most of the world’s olive oil is produced. Olive oil is widely known as the main source of fat in the Mediterranean diet, being related to several beneficial effects on human health, due to its balanced fatty acid composition and antioxidant properties [1]. Therefore, in the last years, there has been an observed increase in olive oil consumption and demand all over the world [2]. Virgin olive oil (VOO) is mainly constituted by triacylglycerols, besides other minor compounds, such as free fatty acids, glyceridic compounds and unsaponifiable constituents [3,4]. Among the glyceride fraction, olive oil presents a high content of unsaturated fatty acids, namely monounsaturated (MUFAs) and polyunsaturated (PUFAs) fatty acids, and a minor amount of saturated fatty acids (SFAs) [2,3]. The most abundant fatty acids are the MUFA oleic acid (C18:1), PUFA linoleic acid (C18:2) and the SFAs palmitic (C16:0) and stearic (C18:0) acids [3]. Regarding minor compounds, they represent about 2% of olive oil composition, mainly constituted by phenolic compounds, which are well known for their potential as antioxidant, anti-inflammatory and antimicrobial agents [3]. There are a large number of phenolics in VOO belonging to different classes such as phenolic acids, phenolic alcohols, flavonoids, secoiridoids, lignans and hydroxyisochromans [4]. In detail, the most representative phenolic alcohols of VOO are hydroxytyrosol and tyrosol, along with their secoiridoid derivatives oleuropein aglycone, oleacin and oleocanthal [4]. These compounds are mainly formed during olive crushing and malaxation for olive oil production, by the hydrolysis of secoiridoid glycosides, namely oleuropein, demethyl oleuropein and ligstroside, the most abundant secoiridoids of the olive fruit [4]. Regarding the other classes of phenolic compounds, verbascoside is the most predominant hydroxycinnamic acid of olive fruits, while the main phenolic acids are caffeic, chlorogenic, vanillic, syringic, *p*-coumaric and gallic acid, which are found in very small amounts in VOO. Among the flavonoid class, flavonol glycosides such as luteolin-7-*O*-glucoside, apigenin-7-glucoside and rutin are the most abundant flavonoids of olive fruits, while luteolin and apigenin are more frequently described for VOO [5]. Despite the general chemical composition of olives and oil, the concentration of fatty acids and polyphenols may vary depending upon different factors, such as the olive cultivar, maturation stage, harvest time, storage conditions, geographical origin and environmental factors [3,5].

Considering the predicted scenarios of climate change, the Mediterranean region will face a substantial shift in precipitation patterns and rises in temperature [6]. The increasing threat of climate change is already having a considerable impact on agricultural production [7]. Particularly in the olive tree, high temperatures and water deficits have been shown to be critical to the fruit development, maturation, yield, oil accumulation, phenolic concentration and sensorial properties of oil [7,8]. On the other hand, agriculture is one of the sectors that most contributes to greenhouse gas emissions, and globally, the intensive use of conventional practices of soil management, such as tillage, has resulted in cases of severe deterioration in soil structure [9]. Tillage is an ancestral method of soil mechanical manipulation to avoid weed competition for water and nutrients, which gives rise to a large area of bare soil susceptible to erosion processes, one of the principal causes of land degradation in the Mediterranean area [10]. In order to mitigate these negative impacts, alternative practices of soil management have been developed. No-tillage practices including cover cropping with leguminous species and soil amendment with natural elements, such as zeolites, are some of the strategies of sustainable soil management that have been studied in recent years [11,12]. Cover cropping avoids periods of bare soil and it is considered an effective way to reduce soil erosion and nitrate leaching and to increase organic matter, soil organic carbon and water infiltration [10]. In olive groves, the use of cover crops as a soil management practice has shown its effectiveness in reducing erosion, increasing biodiversity, improving soil properties and increasing the yield and physiological performance of olive trees [13,14]. In turn, zeolites are crystalline hydrated aluminosilicates, characterized by an infinite three-dimensional structure identified by interconnected cavities or cages [15]. Their particular structural properties such as a high cation exchange capacity (CEC), water and nutrient holding capacity, infiltration rate, adsorption capacity and hydraulic conductivity, determine their wide range of applications in several industries [15,16]. In agriculture, natural zeolites are being used as natural inorganic soil conditioners, slow-release fertilizers and heavy metal removers [15]. The use of zeolites in olive orchards has already been evaluated by some authors, with a reported positive effect on plant growth, leaf relative water content, soil water and nutrient availability [17,18]. Despite the numerous advantages of soil management with cover crops and zeolites for soil quality and structure, little is known about their influence on the olive fruit, VOO quality and fatty acid and phenolic composition. The aim of this study was to evaluate and compare the effects of conventional tillage (T) practice with two sustainable soil management strategies, a leguminous cover crop (LC) and the combined use of natural zeolites with the leguminous cover crop (ZL), on the olive fruit and VOO yield, quality, fatty acid and phenolic composition, under rainfed conditions. As far as we know, the present study is the first reporting on the combined use of leguminous cover crops with zeolites as a sustainable strategy and on its effects on the fatty acid profile and phenolic composition of olive fruits and VOO.

## 2. Results and Discussion

### 2.1. Effects on Crop Yield, Fruit Biometric Traits and Maturation Index

Sustainable soil management practices have been described as a method of managing agroecosystems for improved and sustained productivity, increased profits and food security, while preserving and enhancing the resource base and the environment [19]. Our results showed that both the LC and ZL treatments significantly increased the yield of olive trees in 2018 when compared to T, whereas in 2019, the differences were not statistically significant (*p* = 0.083), despite being verified as the same tendency (Table 1). Regarding cumulative yields, LC and ZL trees presented an enhancement of 31.6% and 35.5% over trees submitted to tillage. The increase in crop yield by leguminous cover crops in relation to soil tillage in similar environmental conditions has previously been reported [14]. Conservation agriculture practices, such as leguminous cover crops and zeolites, increase yield through the improvement of several biological, physical, chemical and hydrological soil properties, while tillage’s negative impact on productivity is related to its harmful effects on root growth and poor water and nutrient use efficiencies [19]. Among other consequences, tillage increases the disruption of soil aggregates and decreases soil macroporosity, which are critical for root penetration, water movement and gas diffusion, as well as reducing the infiltration rate due to soil crusting [20], thereby creating more water and nutritional constraints for trees.

Besides their crop yield, the study of the biometric characteristics of olive fruits is also of interest. Along with some biometric parameters such as fruit size, flesh content and flesh/pit ratio being widely used to evaluate the quality of table olives, they can also be used as indicators of fruit growth and development [21] and have an influence on oil yield. Fruit growth is determined by the processes of cell division and expansion. During the last phases of olive fruit growth, the cell number and the rate of cell expansion appears to play an important role in the availability of fruit assimilates and, thus, in mesocarp composition, which is one of the most important commercial criteria of olives [22]. Table 1 shows the olive’s biometric traits. Fruit fresh weight (FW), pulp FW, longitudinal length, equatorial length and fruit moisture varied significantly between treatments, showing, in general, higher LC values, mainly in 2019. In addition, fruit traits also varied significantly with the harvest year. Fruits from the 2019 harvest showed higher values of fruit FW, pulp FW, pit FW, longitudinal length, equatorial length and pulp/pit ratio, in a strictly negative association with the crop yield. In the same way, a lower crop load was the main determinant for the higher fruit maturation index (MI) in 2019, which ranged from the lowest value of 2.57 in T to 3.67 in LC and 3.81 in the ZL treatment, while in 2018, there were no significant differences among treatments, with values of 2.56, 2.95 and 2.37, respectively. A similar influence of the fruit load on the fruit weight and maturation index has been reported previously [20,23,24]. Nonetheless, in 2019, it is noteworthy that the more advanced ripening stage of ZL and LC fruits, in spite of a tendency to a higher crop yield, probably related with the superior availability of assimilates that caused earlier fruit ripening [25].

### 2.2. Influence on Phenolic Compound Concentrations and Antioxidant Capacity of Fruits and Olive Oil

**Figure 1:**
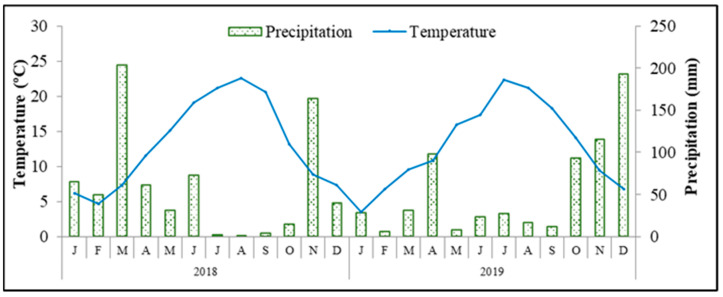
Average monthly temperature and precipitation conditions recorded during the experimental period in the weather station in Paradela, close to the experimental plot.

Table 2 shows the influence of soil treatments on the olive fruit’s phenolic compounds and antioxidant capacity. In 2018, the concentrations of ortho-diphenols and flavonoids as well the total antioxidant capacity (TAC) were significantly higher in T fruits, while in 2019, no significant differences among treatments were observed. Thus, the values of MI presented before did not appear to be the most relevant factor to explain these responses. We are confident that the results from 2018 are defended by the carbon-nutrient balance hypothesis [26]. Under conditions of more water and nutritional constraints, as discussed before, the T trees’ yield and growth were more reduced than their photosynthesis, resulting in a higher accumulation of nonstructural carbohydrates which could be diverted into an enhanced production of defense carbon-based metabolites, such as phenolics compounds. Moreover, this behavior was stimulated by additional stressful conditions in 2018, resulting from low precipitation and high average and maximum temperatures between July and October, which exacerbated the oxidative stress, promoting the biosynthesis of that group of antioxidant compounds. A similar influence of climate variables on the accumulation of phenolics in olive fruits has been reported previously [27]. Moreover, the influence of treatments and the harvest year on olive oil phenolics and TAC is shown in Table 3. In 2018, total phenols (TP), *ortho*-diphenols and TAC were higher in the T treatment group, in a close association with the fruit data, while in 2019, these values were generally higher in the LC olive oil. Meanwhile, the concentration of flavonoids significantly varied with the harvest year, being higher in 2018, while the concentration of *ortho*-diphenols and TAC was superior in 2019. As can be seen in Figure 1 and Table 4, during the fruit development period, the average temperature was higher and the cumulative precipitation was lower in 2018 than in 2019, especially in October, close to harvest. The accumulation of phenolic compounds is a well-known adaptive mechanism in the olive tree against low water availability, during which they play key functions as antioxidants in stressing plants by inhibiting the generation of and reducing reactive oxygen species [1]. Interestingly, in spite of similar levels in fruits, higher values of TP, *ortho*-diphenols and TAC were observed in LC than in T olive oil in 2019 and the intermediate amounts of *ortho*-diphenols and TAC were also superior in the ZL oil than from the tillage treatment. Such data mean that a high percentage of phenolic compounds was transferred from the olive pulp to the oil in these two treatments, namely in LC, related to changes in enzymes’ activities during the pressing and malaxation steps and/or changes in the transference of specific phenolics presented in the fruits and olive stones and lignans after whole olive fruit crushing and malaxation [28]. This contradicts the deduction that the concentration of phenols in fruits is fully associated with the concentration of phenolics in oil.

### 2.3. Effects on Polyphenolic Composition of Fruits and Olive Oil

Table 5 shows the fruit polyphenolic composition under the effect of soil treatments and the harvest year, which included phenolic alcohols, phenolic acids, flavonoids, secoiridoids and hydroxycinnamic acids. A total of 14 phenolic compounds was identified in the olives. TP concentrations were obtained by adding up the amounts of individual phenolics. All the samples showed the same chromatographic profile, with only variations observed in polyphenolic concentration between treatments. In general, the obtained values for each phenolic compound are in accordance with those found in the literature [8,29]. The predominant phenolic compound founded was oleuropein, usually described as the major bioactive compound in olive fruits [30,31]. During maturation and due to olive processing for olive oil production, the concentration of oleuropein tends to decline as a result of enzymatic reactions, which converts it into derivatives, such as hydroxytyrosol and tyrosol [31]. The richness of olives in oleuropein and its degradation products is particularly relevant considering their nutritional and health benefits due to their cardioprotective, anti-inflammatory and anticancer properties [32]. Generally, stressful conditions are reported to increase the accumulation of polyphenols in olives, promoting the activity of l-phenylalanine ammonia-lyase, the key enzyme in phenolic biosynthesis [8,33]. This appears to make sense with the results of 2018, as a significant increase in TP was observed for the T treatment, which was mainly related to the increase in hydroxytyrosol, chlorogenic acid, luteolin-3,7-di-*O*-glucoside, quercetin-3,7-di-*O*-glucoside and rutin, confirming the results of the *ortho*-diphenol and flavonoid concentrations. Interestingly, in 2019, our results showed that ZL treatment promoted an increase in oleuropein, rutin, hydroxytyrosol, verbascoside, epicatechin, tyrosol and the minority compounds apigenin-7-*O*-glucoside and quercetin-3,7-di-*O*-glucoside, which resulted in a higher content of TP. Meanwhile, in 2018, despite the lower TP levels, ZL-treated olives presented superior concentrations of verbascoside, epigallocatechin and caffeic acid relative to the other treatments, while LC olives showed the highest gallocatechin content. Thus, the combined use of zeolites with leguminous cover crops might have an influence on some soil–plant interaction processes that influence fruit polyphenolic compositions. Besides the increase in major phenolic compounds by ZL in 2019, other variations on the polyphenolic profile occurred between treatments, namely the higher contents of gallocatechin, luteolin-3,7-di-*O*-glucoside and apigenin in the LC fruits and the superior concentration of epigallocatechin in the T fruits.

On the other hand, the olive fruit phenolic composition was also significantly affected by the harvest year. In 2018, higher levels of hydroxytyrosol, tyrosol, verbascoside, oleuropein, gallocatechin, quercetin-3,7-di-*O*-glucoside, rutin and apigenin-7-*O*-glucoside were observed, whereas in 2019, above all, a higher concentration of luteolin-3,7-di-*O*-glucoside was observed.

Olive oil’s phenolic profile is given in Table 6. A total of 14 compounds were identified in olive oils, but some of them were different from those detected in olive fruits. The obtained values for each phenolic compound were slightly lower than those found for olive oil in other studies [29,34]. As observed for the olive fruits, in 2018, a higher concentration of TP was obtained in the T olive oils. On the other hand, in 2019, LC treatment showed superior TP levels, contrarily with what was found in the olive fruits. These results are also in accordance with those obtained by the Folin-Ciocalteu determination. In the 2018 harvest, the higher TP levels in T olive oils was mainly due to an increase in hydroxytyrosol, 3,4-DHPEA-EDA, oleuropein and apigenin contents, while in 2019, the higher concentration of TP verified in the LC treatment was due to the enhancement of hydroxytyrosol and luteolin-7-*O*-glucoside. Meanwhile, although the trend observed in the concentration of TP in fruits from the 2019 harvest was not verified in the oil, in general, the oils from the ZL treatment presented consistently higher levels of DHPG, tyrosol, caffeic acid and verbascoside in both years. Apart from the differences in climate conditions between the years, as previously mentioned, the variation in the concentrations of phenolics in fruits and olive oil can be related to other factors, namely crop load, fruit maturation stage and fruit moisture. Higher concentrations of total phenolic compounds under tillage in 2018, associated with lower crop loads, may be seen as a result of limited competition for sugars between sink organs, which leads to an increase in the amount of sugars transferred into olives. Similar results were observed in other works [35,36,37,38]. On the other hand, the higher secondary metabolite concentrations in legume treatments in 2019, a year without significant differences in crop yield, may have resulted from the opportunity to take advantage of the excess pool of carbon to build cost-free, carbon-based, secondary metabolites, as pointed out by others [38,39,40]. Furthermore, the higher MI of LC and ZL fruits may also be relevant since in this genotype, Ferro et al. (2020) [41] found higher phenolic concentrations in fruits with similar maturation stages to the present study. Contrasting results in the concentration of phenolic compounds between years in this species were also reported by Monasterio et al. (2021) [42]. Meanwhile, although in general the concentration of phenolic compounds in olive oil increases with a decrease in fruit moisture within the range of 60–50% [42], our data did not support this assumption as LC oil from 2019 presented higher levels, in spite of the highest fruit moisture (Table 1), and the lower concentration of phenolics in the fruits, when compared with olives from the ZL treatment, suggesting a superior phenolic transfer yield.

The influence of the harvest year on the phenolic composition also deserves to be highlighted. In fact, in the olive oil samples from 2018, higher concentrations of hydroxytyrosol, hydroxytyrosol acetate, oleuropein aglycone, caffeic acid, 3,4-DHPEA-EDA, verbascoside, oleuropein, rutin and apigenin were found, whereas in the olive oil samples from the 2019 harvest, superior levels of DHPG, tyrosol, luteolin-7-glucoside and apigenin-7-*O*-glucoside were observed. Changes in the phenolic composition between crop seasons have also been verified by other researchers [43,44,45].

**Figure 2:**
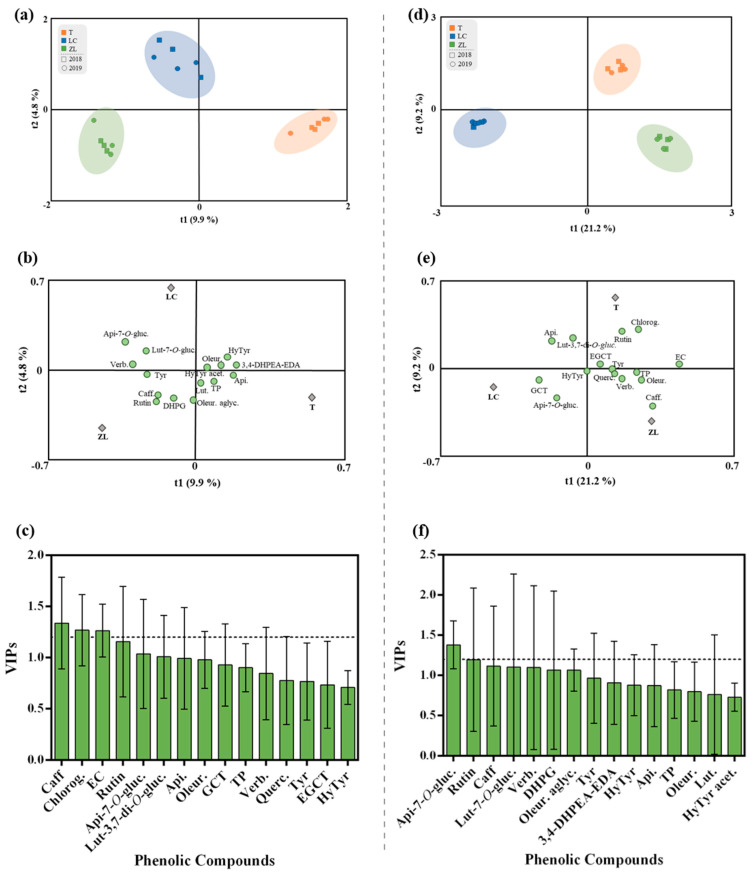
(**a**–**c**) OPLS-DA of olive fruit and (**d**–**f**) olive oil phenolic compounds, irrespective of the year. (**a** and **d**) Scores plot and (**b** and **d**) loadings plot of the first two factors of the OPLS-DA model built with the phenolic profile of the olive oil according to the soil treatment. (**c**,**f**) Phenolic compounds ranked by VIP scores. Caffeic acid (Caff), chlorogenic acid (Chlorog), rutin, luteolin-3,7-di-*O*-glucoside (Lut-3,7-di-*O*-gluc.), epicatechin (EC), apigenin-7-*O*-glucoside (Api-7-*O*-gluc), apigenin (Api), oleuropein (Oleur), gallocatechin (GCT), total phenols (TP), verbascoside (Verb.), quercetin-3,7-di-*O*-glucoside (Querc.), tyrosol (Tyr), epigallocatechin (EGCT), hydroxytyrosol (HyTyr), luteolin-7-*O*-glucuside (Lut-7-*O*-gluc.), 3,4-dihydroxyphenylglycol (DHPG), oleuropein aglycone (Oleur. aglyc.), dialdehydic forms of decarboxymethyl elenolic acid linked to hydroxytyrosol (3,4-DHPEA-EDA), luteolin (Lut.), hydroxytyrosol acetate (HyTyr acet.).

In order to explore the impact of the different agricultural practices on the phenolic composition of olive fruits and olive oil irrespective of the harvesting year, orthogonal partial least squares-discriminant analysis (OPLS-DA) was used. The OPLS-DA integrated an orthogonal signal correction filter to separate the variations in the data that are related to the prediction of a quantitative response from the variations not related or orthogonal to the prediction. The OPLS-DA supervised approach was performed to better account for markers of the differences in the observed phenolic profile (Figure 2). The class prediction model clearly differentiated the olive fruits according to the different agricultural practices confirming that the actual phenolic profile of olive fruits was imposed by the different agricultural practices. In this regard, the quality parameters of the OPLS-DA model were excellent with a very high R2Y and Q2Y (0.99 and 0.98, respectively). No outlier samples were observed by Hotelling’s T2, whereas both CV-ANOVA and the permutation test (given as Supplementary Materials; Table S1 and Figure S1) showed a more than adequate degree of validation. Afterwards, the variables’ importance in the projection of the OPLS-DA model was evaluated (VIP analysis), in particular considering the VIP scores for each phenolic compound analyzed. The VIP score summarizes the contribution a variable makes to the model, and it is calculated as a weighted sum of the squared correlations between the OPLS-DA components and the original variables. Phenolic compounds with the highest VIP score (>1.2) were caffeic acid, chlorogenic acid and epicatechin (Figure 2c).

**Figure 3:**
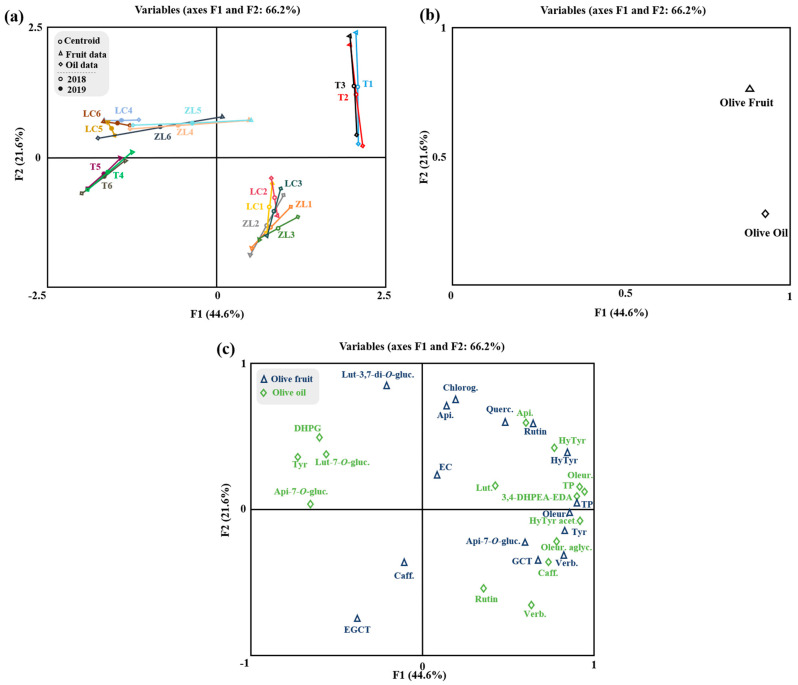
Multiple factorial analysis of phenolic compounds of olive fruit and olive oil. (**a**) Representation of olive samples and clouds; (**b**) representation of groups (tables) of variables; and (**c**) distribution of variables. Centroid (○); olive fruit data (∆); olive oil data (◊); centroids respective to samples from 2018 (○) and 2019 (●). Apigenin-7-*O*-glucoside (Api-7-*O*-gluc.), caffeic acid (Caff), luteolin-7-*O*-glucuside (Lut-7-*O*-gluc.), luteolin-3,7-di-*O*-glucoside (Lut-3,7-di-*O*-gluc), rutin, verbascoside (Verb.), 3,4-dihydroxyphenylglycol (DHPG), oleuropein aglycone (Oleur. aglyc.), tyrosol (Tyr), dialdehydic forms of decarboxymethyl elenolic acid linked to hydroxytyrosol (3,4-DHPEA-EDA), hydroxytyrosol (HyTyr), apigenin (Api.), total phenols (TP), oleuropein (Oleur.), luteolin (Lut.), hydroxytyrosol acetate (HyTyr acet.), epigallocatechin (ECGT), epicatechin (EC), gallocatechin (GCT), chlorogenic acid (Chlorog.), quercetin-3,7-di-*O*-glucoside (Querc.).

The OPLS-DA relative to the olive oil phenolic compounds is represented in Figure 2d–f. The olive oils obtained from the different soil treatments were clearly discriminated by the class prediction model. The quality of the OPLS-DA model was validated by the values of the R2Y and Q2Y parameters (0.95 and 0.85, respectively), which demonstrates the potential usefulness of OPLS-DA. No outlier samples could be observed by Hotelling’s T2, and both CV-ANOVA and the permutation test (given as Supplementary Materials; Table S2 and Figure S2) showed an adequate degree of validation. According to the VIP results, apigenin-7-*O*-glucoside and rutin were the most influential variables in the discrimination of olive oils between treatments. On the other hand, a multifactorial analysis (MFA) was applied to the fruit and oil phenolic compound data (Figure 3). MFA is a useful method in analyzing several tables of variables simultaneously and in studying the relationship between the observations, variables and tables. Figure 3a represents the olive fruit and oil samples and clouds. The coordinates of the group of variables (tables) were displayed and used to create the map of tables (Figure 3b). From the variable map, it can be concluded that for the first factor, both groups of variables contributed almost equally (89.6% and 91.5% for the fruit and oil, respectively), while for the second factor, fruit variables contributed 64.2% and oil variables contributed 23.4%. Moreover, according to the Lg measurements, the first axis corresponded to a direction of very significant inertia for each group (1.422 and 1.124, respectively, to olive fruit and olive oil variables). Olive fruit was positively correlated with both factors (F1 and F2). This loading could be related to the correlation of olive fruit TP, hydroxytyrosol, rutin, quercetin-3,7-di-*O*-glucoside, chlorogenic acid, apigenin and epicatechin. Olive oil was positively correlated with F1, with a positive correlation between this factor and the olive oil phenolic compounds hydroxytyrosol acetate, oleuropein aglycone, caffeic acid, verbascoside and rutin also being observed. Overall, these results are in accordance with the literature, whereby the phenolic compounds that were positively correlated with the olive fruit and olive oil are described as the most frequent compounds found either in olive fruit or olive oil [4].

It is well known that olive crushing and malaxation processes inevitably change the profile of phenolic compounds and, therefore, both the organoleptic and antioxidant properties of olive oil [46]. MFA shows that some phenolic compounds are highly correlated between the olive fruit and olive oil (Figure 3c), as is the case for hydroxytyrosol. Moreover, the oleuropein and tyrosol from olive fruits were highly correlated with the hydroxytyrosol acetate and oleuropein aglycone from olive oil. In fact, these compounds are biosynthetically related, belonging to the same metabolic pathways. Thus, tyrosol and its derivatives may be converted to hydroxytyrosol and its derivatives and vice versa. In turn, oleuropein aglycone is derived from the deglycosylation of oleuropein during olive oil processing [46].

**Figure 4:**
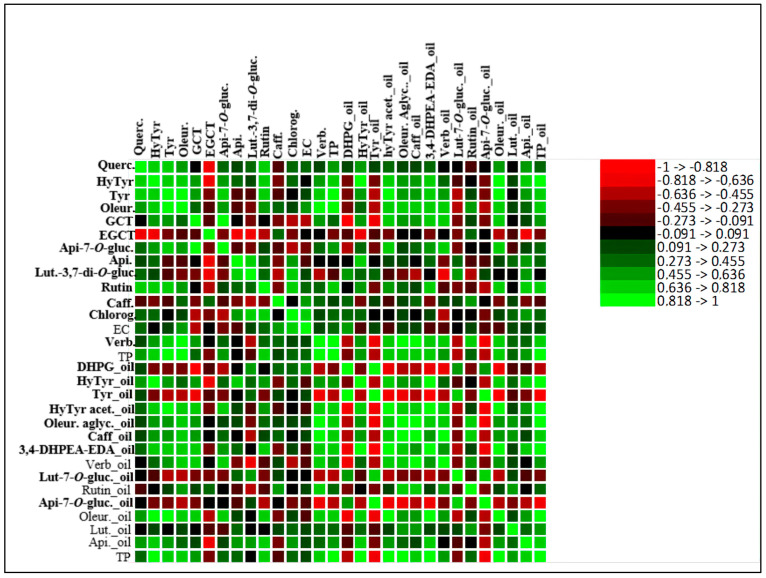
Heatmap of the correlation matrix between olive fruit phenolic compounds and olive oil phenolic compounds. Apigenin-7-*O*-glucoside (Api-7-*O*-gluc.), caffeic acid (Caff), luteolin-7-*O*-glucoside (Lut-7-*O*-gluc.), luteolin-3,7-di-*O*-glucoside (Lut-3,7-di-*O*-gluc), rutin, verbascoside (Verb.), 3,4-dihydroxyphenylglycol (DHPG), oleuropein aglycone (Oleur. aglyc.), tyrosol (Tyr), dialdehydic forms of decarboxymethyl elenolic acid linked to hydroxytyrosol (3,4-DHPEA-EDA), hydroxytyrosol (HyTyr), apigenin (Api.), total phenols (TP), oleuropein (Oleur.), luteolin (Lut.), hydroxytyrosol acetate (HyTyr acet.), epigallocatechin (ECGT), epicatechin (EC), gallocatechin (GCT), chlorogenic acid (Chlorog.), quercetin-3,7-di-*O*-glucoside (Querc.).

On the other hand, some phenolic compounds, such as tyrosol and DHPG from olive oil, did not show a correlation between the olive fruit and olive oil, which may indicate that their presence in the olive oil did not occur by transference, but probably as a result of chemical or enzymatic reactions. The detailed correlations between all the phenolic compounds of fruit and oil are given by the correlation matrix in Figure 4.

To better understand the contribution of the total phenolic composition of olive fruits on olive oil, a correlation analysis was performed (Figure S3, Supplementary Materials). The correlation between the olive fruit and olive oil total phenolic compounds showed an r value of 0.832 and an r^2^ value of 0.692, indicating a positive correlation between these parameters and confirming that about 69.2% of the variation in the olive oil phenolic composition was provided from the olive fruits. Therefore, the implementation of practices that promote the quality of olive fruits is crucial, since, as was demonstrated, the quality of VOO is closely related to the quality of olive fruits.

### 2.4. Influence on Olive Fruit Fat Content and Fatty Acid Profile

Beyond the olive tree genotype and the incidence of pests and diseases, the total fat content and fatty acid composition are influenced by many factors, including olives’ ripening stage, fruit biometry traits (e.g., fruit weight and pulp-to-pit ratio), fruit moisture, crop load and multiple agronomic and environmental conditions that affect, among other aspects, the plant water, nutritional status and light environment around the canopy [1,8,42,47,48,49,50]. Table 7 shows higher total fat content in both the LC and ZL treatments in 2018, while in 2019, the behavior was diametrically opposite.

The conjugation of these results with the previously presented traits suggests that during this experiment, the MI, fruit weight, pulp-to-pit ratio, fruit moisture and crop yield had a minor influence on fat content. We consider these the main reasons to justifying the lower fat content in fruits from the T treatment in 2018: (1) the precursor to the biosynthesis of fatty acids is acetyl-CoA, derived from a catabolism of sugars [51]; (2) the lower net photosynthesis of T trees ranged from –3% to –36% during the fruit growing season due to higher water and nutrient limitations, as in previous studies [14,52]; and (3) in a hotter and drier year, the sugars were more diverted towards protective mechanisms (e.g., osmotic adjustment, antioxidant activity, leaf structure protection traits) and to respiratory activity and not so much towards fat synthesis. Meanwhile, in 2019, with fewer environmental constraints, plants presented less investment in defense mechanisms and more photoassimilates were channeled per unit of fruit produced to the synthesis of olive oil. It is also likely that the lower vegetative growth of T plants, as confirmed by the reduction (15 to 18%) in pruning weight, may have been relevant, given that a greater proportion of fruits was exposed to more solar irradiation. Olive fruits located in zones of the canopy that are more exposed to solar radiation, such as the top and middle–outer, produce more oil than fruits located in shadow zones, such as the lower parts and inner zones of the trees [48,53].

Regarding fruit fat composition (Table 7), the most abundant FAME of olive oil was oleic acid (C18:1), ranging between 74.5% to 77.4%, followed by palmitic acid (C16:0), between 12.7% up to 14.7%, and linoleic acid (C18:2), from 5.0% to 8.4%. The obtained values accounted for 95.8% to 98.2% of the total fatty acid composition, being in accordance with those found in the literature [8,45,54]. At the same time, all percentages of fatty acids fell within the recommended ranges for EVOO set by EU Regulation (Commission Implementing Regulation, 2019/1604) [55]. The fatty acid profile varied, often significantly, between treatments over the years, but without a clear trend, except for linoleic acid which presented lower amounts in ZL fruits in both years, namely when compared to conventional tillage, resulting in inferior PUFAs and a higher oleic/linoleic acid ratio in the ZL treatment. Meanwhile, in 2018, palmitoleic acid was higher in T than in LC fruits, whereas both cover crop treatments exhibited superior levels of oleic acid and, thus, MUFAs than in the T treatment. On the other hand, in 2019, ZL presented higher levels of palmitic acid and SFAs and an inferior UFA/SFA ratio than in the T olives.

**Figure 5:**
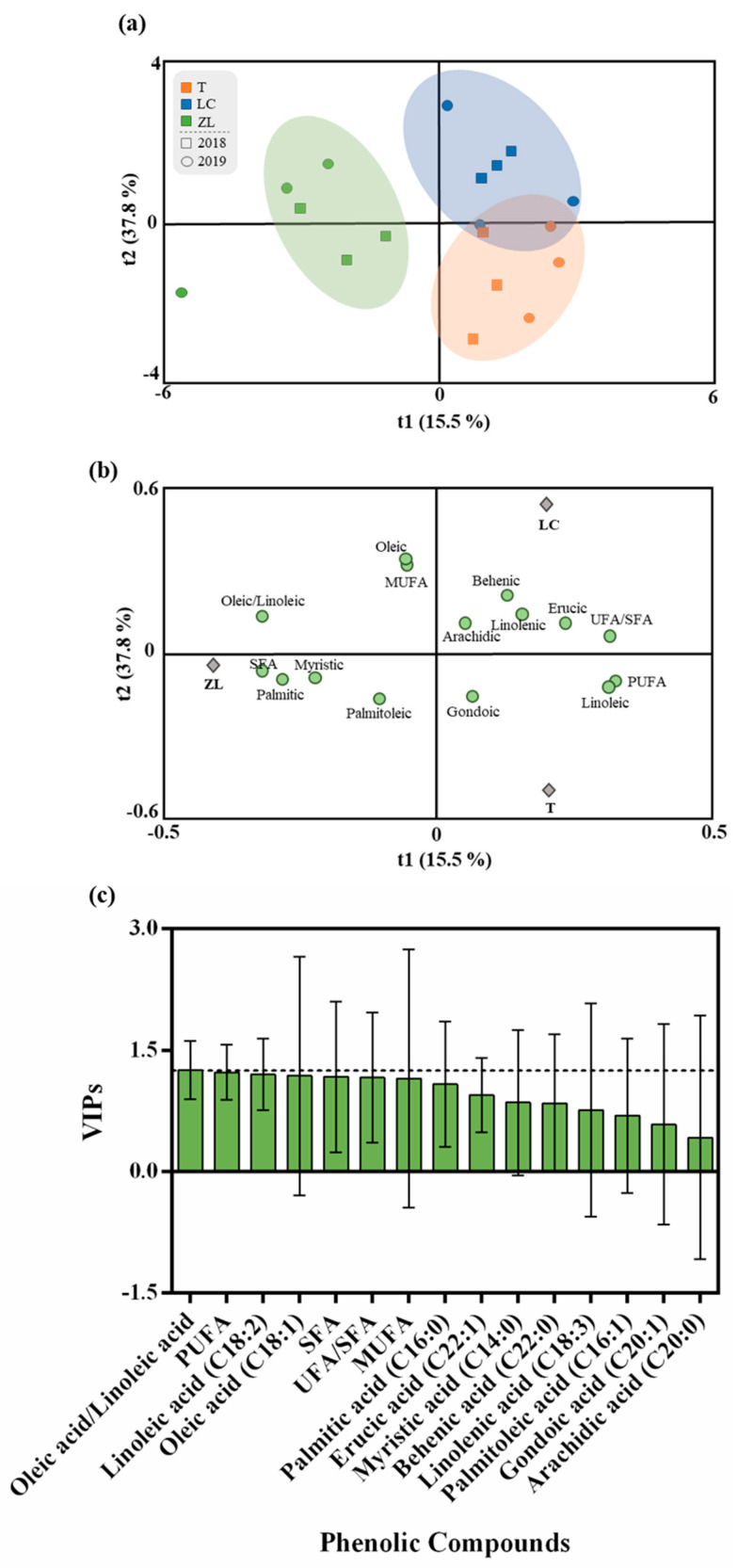
(**a**) Scores plot and (**b**) loadings plot of the first two factors of the OPLS-DA model built with the phenolic profile of the olive oil according to the soil treatment. (**c**) Phenolic compounds ranked by their VIP scores.

Similarly, to what was performed for phenolic compounds, an OPLS-DA was made (Figure 5) in order to understand the impact of the different soil treatments on the fatty acid profile of the olive fruits. The results show that with a small exception for the ZL treatment, the other soil treatments were not very differentiated from one another, showing little influence of soil treatment on the fruit fatty acid profile. Thus, the quality parameters of the OPLS-DA model were low with values of 0.66 and 0.48 for the R2Y and Q2Y, respectively. The CV-ANOVA and the permutation test referent to these analyses are given in the Supplementary Materials (Table S3 and Figure S4). Despite these small differences, according to the VIP results, the variables that most contributed to discrimination were oleic acid, PUFA and linoleic acid. Despite the fluctuations verified in the fatty acid profile between treatments, a clear trend was observed for a higher oleic/linoleic ratio in the ZL fruits relative to the other two treatments, namely for fruits produced in tilled soil. A higher oleic-to-linoleic acid ratio due to the use of legume cover crops, when compared to soil tillage, was also reported by Sastre et al. (2016) [24]. Thus, considering this ratio and the low proportion of PUFA, ZL oil may be considered better from a nutritional and technological point of view, with higher oxidative stability and, therefore, a superior shelf life and thermal performance [33,56,57].

### 2.5. Influence on Oil Quality Parameters

The commercial quality of the extracted oils was affected by the soil management system, but with greater magnitude in the first year of the experiment (Table 8). Overall, the results show that all oils from 2018 were classified in the “extra virgin” category, showing low mean values of free acidity (FA; <0.8%), peroxide index (PI; <20 mEq O_2_ kg^−1^), K270 (<0.22), K232 (<2.5) and ΔK (<0.01) [58], while in 2019, a poorer quality of olive oils was observed, considering the increase in K232 slightly above the threshold imposed by the EU regulation. In 2018, in general, oils from legume cover crops, especially when combined with zeolites, showed lower values of FA, PI, K232, K270 and ∆K. While FA measures the hydrolytic breakdown of triglycerides to di- and monoglycerides, leading to fatty acid release, PI measures the release of peroxide compounds arising from primary oxidation and spectrophotometric values, and K232 and K270 measure conjugated dienes and trienes and their secondary oxidation products [59]. The highest values obtained in the T olive oils suggests a higher level of hydrolytic breakdown, damage and oxidation, than in the other soil treatments. These responses do not tend to be associated with olives’ maturity index or with phenolic concentration but with the fatty acid profile, particularly with the oleic/linoleic and MUFA/PUFA ratios, as described before, probably due to a higher activity of oleate desaturase during the oil accumulation period in fruits from the tillage plot. Having scarce information about the influence of legume cover crops on the commercial quality indices of olive oils, previous studies have revealed a similar pattern of free acidity response [24,60], while no significant effects of legumes were detected in the peroxide index, K232, K270 or ∆K [24]. Meanwhile, the application of zeolites increased the oleic/linoleic ratio and ameliorated the oil quality indices FA, PI and K232, when compared with the single use of NPKB fertilizers in a rainfed olive orchard [59]. On the other hand, in 2019, the free acidity response was reversed, showing that the T olive oil had the lowest value, while for the rest of the evaluated parameters (i.e., primary oxidation (PI, K232), advanced oxidation (K270), hydrolytic alteration (acidity)), no influence of soil management treatment was found. We believe that the most decisive factor for the FA response was the higher fruit maturation index of both legume cover crops, highlighting the importance of earlier harvesting to obtain optimum quality oils [61].

## 3. Materials and Methods

### 3.1. Site Description, Cultural Practices and Plant Material

The field experiment was carried out in Suçães, Mirandela (41°29’ N, 7°15’ W), in northeastern Portugal, from September 2016 to December 2019. This region has a typical Mediterranean climate characterized by a warm and temperate climate with dry and warm summers. The detailed climatic characteristics are shown in Table 9. The commercial orchard was 27-years-old of “cv. Cobrançosa”, planted at a frame of 7 × 7 m and rainfed managed. The soil was of a Leptosol derived from schist, sandy loam texture, pH (H_2_O) 5.1, with low organic matter (Walkley-Black) content (13.0 g kg^−1^) and with low and high levels of extractable (Egner-Riehm) phosphorus (30.5 mg kg^−1^) and potassium (79.8 mg kg^−1^), respectively. After a long period of soil management by tillage since the orchard was planted, the farmer changed the system in 2006 by using glyphosate in a single application in mid-spring. The fertilization program followed in the orchard in the previous years consisted of a compound NPK fertilizer (10:10:10) applied annually beneath the trees’ canopy at a rate corresponding to 60 kg ha^–1^ of N, P_2_O_5_ and K_2_O, supplemented with 2 kg B ha^–1^ as borax. Shortly before the trial started, 1000 kg ha^–1^ of lime (88% CaCO_3_ and 5% MgCO_3_), 150 kg ha^–1^ of potassium chloride (60% K_2_O) and 200 kg ha^–1^ of superphosphate (18% P_2_O_5_) was applied. After the trial started, no fertilizers were added to the trees. Moderate pruning, performed annually in the winter resting period, removed around 20% of foliage.

### 3.2. Ground Management and Experimental Layout

The orchard selected for this experiment is a homogeneous plot regarding its slope (~2%) and general soil and tree characteristics. It was divided into three adjacent experimental subplots of 1800 m^2^ each (four rows of 60 m length), with one to receive conventional tillage (T), consisting of two tillage passes per year in spring using a cultivator, and two subplots to receive a cover crop consisting of a mixture of self-reseeding annual legume species and cultivars. The cover crop, sown in September 2016 at a rate of an eleventh of that recommended by each species if seeded alone in pure culture, was composed of a mixture of eleven annual pasture legumes (*Ornithopus compressus* L. cv. Charano; *Ornithopus sativus* Brot. cvs. Erica and Margurita; *Trifolium subterraneum* L. *ssp. subterraneum* Katzn. and Morley cvs. Dalkeith, Seaton Park, Denmark and Nungarin; *Trifolium resupinatum* L. *ssp. resupinatum* Gib and Belli *cv.* Prolific; *Trifolium incarnatum L.* cv. Contea; *Trifolium michelianum* Savi cv. Frontier; and *Biserrula pelecinus* L. cv. Mauro). The seeds were broadcast by hand and incorporated with a shallow cultivator and a roller. During May, the above ground biomass of the cover crop subplots, dominated by crimson clover, were destroyed with a rotary slasher and left on the ground as a mulch. Then, one subplot received the surface application of natural zeolites (1500 kg ha^–1^) combined with mulch, whereas the other subplot had only the plant debris left on the soil surface, hereafter designated as zeolites with leguminous cover crop (ZL) and leguminous cover crop (LC) treatments, respectively. The zeolites’ (ZeoCat, Barcelona, Spain) main properties are shown in Table 9. All assessments were performed in nine trees (three replicates, each composed of three trees) within the inner two rows of trees of each subplot to avoid border effects.

### 3.3. Yield and Olive Sampling

Olive trees were harvested on 27 October 2018 and 7 November 2019 by a trunk shaker head, which detaches the olive fruits and collects them by an associated inverted umbrella system. The yield was weighed per groups of three trees. Olive fruit samples of each group were collected for biometric and MI analyses, olive oil extraction and biochemical analysis. The analysis of fruit biometric traits and MI, as well as the olive oil extraction, were performed immediately after harvest, while the olive samples for biochemical analysis were pitted and stored at −80 °C for posterior determination.

### 3.4. Fruit Biometric Traits and Maturation Index

Groups of 50 olive fruits from each replicate were randomly selected to evaluate the fruit, pulp and pit FW, dry weight (DW) and longitudinal and equatorial length. Fruit moisture and pulp/pit ratio parameters were calculated according to the following formulas:

$$\mathrm{Fruit}\;\mathrm{moisture}\;\left(\%\right){=((\mathrm{Fruit}}_{\mathrm{FW}}{\;-\;\mathrm{Pit}}_{\mathrm{DW}}{\;-\;\mathrm{Pulp}}_{\mathrm{DW}}{)/\mathrm{Fruit}}_{\mathrm{FW}})\;\times \;100;$$

$$\mathrm{Pulp}/{\mathrm{Pit}\;\mathrm{ratio}}_{\mathrm{FW}}\;{=\mathrm{Pulp}}_{\mathrm{FW}}{/\mathrm{Pit}}_{\mathrm{FW}}.$$

The MI was determined through the classification of 50 olive fruits from each replicate into eight categories based on epidermis and pulp colour (0–7), according to Brito et al. (2021) [62]. The scale started from fruits with an intense green epidermis (MI = 0) and ended with fruits with a black epidermis and totally purple pulp (MI = 7). The MI was calculated as: MI=(a × 0+b × 1+c × 2+d × 3+e × 4+f × 5+g × 6+h × 7)/n, where the letters a–h are the number of fruits in each category and n is the total number of olives assessed.

### 3.5. Fruit Fat Content and Fatty Acid Profile Determination

For olive fruit fat content and fatty acid profile determinations, olive fruits of each replicate were subjected to Folch’s extraction method [63], with some adaptations. To 2 g of lyophilized olive flesh was added 50 mL of Folch’s solution (chloroform:MeOH (3:1) with 75 mg L^-1^ butylated hydroxytoluene), followed by an ultra-turrax mechanical homogenization. The obtained extract was filtered into a separating funnel. After two extractions, the volume was adjusted up to 150 mL with Folch’s solution, followed by the addition of 37.5 mL of NaCl (0.73%). After resting overnight, the organic phase was collected into evaporating flasks and the solvent was completely evaporated on a rotary evaporator at 45 °C. The flask was reweighed after 24 h in a desiccator. The fat content was calculated as follows: $\mathrm{Fat}\;\mathrm{content}\;\left(\%\right){=(W}_{1}{\;-\;W}_{0}{)/W}_{s\;}\times \;100$, where W_1_ is the flask weight after evaporation, W_0_ is the initial flask weight and W_s_ is the initial sample weight. The evaporated content was diluted in 2 mL of *n*-hexane and submitted to a derivatization procedure to promote the conversion of the free fatty acids to their methyl esters. To derivatize, 100 µL of lipid extract were added to 2 mL of MeOH:*n*-hexane (2:1) and placed on ice for the careful addition of 200 µL acetyl chloride. After 60 min at 100 °C on a heating block, 1.5 mL of n-hexane and 6 mL of potassium carbonate 6% was added. Then, the mixture was centrifuged at 1008 *g* for 5 min. The organic phase of each sample was collected and used for the chromatographic analysis on a Trace GC gas chromatograph (Thermo Scientific, Waltham, MA, USA) equipped with a flame-ionized detector and autosampler, fitted with a fused silica capillary column (Supelcowax^®^ 10, with 30 m length × 0.25 mm ID and 0.25 µm film thickness). For the chromatographic analysis, 1 µL of each sample was injected and submitted to a total run of 48 min, programmed to start with an oven temperature of 140 °C during 2 min, followed by a gradient from 140 °C to 220 °C (4 °C min^-1^) and maintained at 220 °C for 20 min. The injector (splitless) and detector temperatures were held at 250 °C. Helium was used as the carrier gas at a flow rate of 1 mL min^-1^. All samples were run in triplicate and the results were expressed in relative percentages for each fatty acid, calculated by the internal normalization of the chromatographic peak area. The fatty acid profile analysis was performed using XCalibur ^TM^ 2.0.7 SP1 Software (Thermo Fisher Scientific, Waltham, MA, USA), by a comparison of the retention times (RTs) of the sample’s peaks with those from the reference standard run under the same conditions. The sample’s fatty acid profile was presented in terms of SFAs (myristic acid (C14:0), palmitic (C16:0), arachidic (C20:0) and behenic (C22:0)), MUFAs (palmitoleic (C16:1), oleic (C18:1), gondoic acid (C20:1) and erucic acid (C22:1)) and PUFAs (linoleic (C18:2) and linolenic (C18:3)).

### 3.6. Olive Oil Extraction and Quality Analyses

The olive oil extraction was performed within 24 h of the olive harvest. The oil was extracted by processing 20 kg of olives from each replicate at the malaxation temperature of 25 °C for 30 min, using olive oil extraction equipment (OLIOMIO 50; Toscana Enologica Mori, Tavarnelle Val di Pesa (FI), Italy). Posteriorly, oils were filtered and placed in dark glass bottles and kept at 4 °C.

The olive oil quality parameters FA, PI, K232, K270 and ΔK were determined according to the European community regulation EEC/2568/91 [64]. The FA and PI were expressed as the percentage of oleic acid per 100 g of olive oil and mEq of O_2_ kg^−1^ of oil, respectively.

### 3.7. Extraction and Quantification of Polyphenolic Compounds from Olive Fruits and Olive Oil

For the olive fruit polyphenolic compound extraction, 30 mL of MeOH:H_2_O (50:50) was added to 2 g of lyophilized olive flesh. After shaking for 30 min at room temperature, the samples were centrifuged at 1000 *g* for 10 min. This step was repeated three times. To remove the fat phase, the mixture was washed twice with 50 mL of *n*-hexane and the organic phase was discarded. The volume was adjusted to 200 mL with MeOH:H_2_O (50:50) [65]. For olive oil polyphenolic compound extraction, 3 mL of oil was used along with 1.25 mL MeOH:H_2_O (70:30) and 1.25 mL *n*-hexane. The mixture was centrifuged for 10 min at 2800 *g*. The lower phase was carefully collected and reserved in a clean flask. This procedure was repeated three times. The final extract was adjusted to 5 mL with MeOH/H_2_O (70:30) [66]. The obtained extracts were used for the quantification analyses of TP, *ortho*-diphenols, flavonoids and total TAC, which were performed as described by Brito et al. (2018) [27]. TP and *ortho*-diphenols were expressed as the mg of gallic acid equivalents (GAE) and flavonoids were expressed as the mg of catechin equivalents (CE) per g of olive flesh DW or kg of olive oil, while TAC was expressed as the mmol of Trolox equivalent (TE) per g of olive flesh DW or kg of oil. All measurements were performed in triplicate.

### 3.8. High-Performance Liquid Chromatography (HPLC) Analysis of Olive Fruits and Oil Polyphenols

To proceed to HPLC analysis, 100 mL of olive flesh methanolic extract was evaporated at 35 °C and redissolved in 2 mL of MeOH:H_2_O (50:50). The phenolic profile was performed by reversed-phase (C18) HPLC using an Ultimate 3000 HPLC system (Dionex Corporation, Sunnyvale, CA, USA), equipped with an Ultimate 3000 pump and column compartment, a WPS-3000 TSL analytics autosampler and a photodiode array detector (PDA-100; Dionex Corporation, Sunnyvale, CA, USA). The compound separation was reached by gradient elution on an ACE 5 C-18 column (250 mm × 4.6 mm) (Advanced Chromatography Technologies, Aberdeen, UK). The eluent was constituted by 0.1% aqueous formic acid (solvent A) and methanol (solvent B). The elution program was characterized by a linear gradient analysis for a total run time of 80 min used as follows: use 5% of solvent B during 2 min, increase to 80% over 68 min, keep isocratic for 8 min, decrease to 5% of solvent B over 2 min and lastly, keep isocratic for 5 min used. The photodiode detector was operated between 200–600 nm and the chromatographic profile was recorded at 280 and 325 nm. The sample volume injected was 50 µL at a flow rate of 0.5 mL min^-1^ and the column temperature was maintained at 30 °C. Quantification was performed with calibration curves with standard (−)-gallocatechin, caffeic acid, hydroxytyrosol, tyrosol, (−)-epigallocatechin, chlorogenic acid, epicatechin, rutin, quercetin-3,7-di-*O*-glucoside, luteolin-3,7-di-*O*-glucoside, apigenin-7-*O*-glucoside, verbascoside, oleuropein and apigenin.

For olive oil polyphenolic HPLC analysis, 5 mL of olive oil methanolic extract was evaporated at 35 °C and redissolved in 2 mL of MeOH:H_2_O (70:30). The phenolic profile was performed by a Thermo Fisher Scientific Vanquish Core HPLC system (Waltham, MA, USA), equipped with a pump, column compartment, autosampler and a diode array detector. The compound separation was reached by gradient elution on a C18 Merck Purospher^®^ STAR, Hibar^®^ C18 column (250 mm × 4.6 mm; particle size 5 μm). The eluent was constituted by 0.1% aqueous formic acid (solvent A) and methanol (solvent B). The elution program was characterized by a linear gradient analysis for a total run time of 100 min used as follows: initiate with 20% of solvent B, followed by an increase to 95% of solvent B over 90 min, keep isocratic for 5 min, decrease to 20% of solvent B over 1 min and lastly, keep isocratic for 4 min. The photodiode detector was operated between 200–600 nm and the chromatographic profile was recorded at 280 and 325 nm. The sample volume injected was 50 µL at a flow rate of 0.5 mL min^-1^ and the column temperature was maintained at 30 °C. Quantification was performed based on calibration curves of standards of hydroxytyrosol, caffeic acid, verbascoside, luteolin-7-*O*-glucoside, rutin, apigenin-7-*O*-glucoside, oleuropein, luteolin and apigenin. All standards were purchased from Sigma-Aldrich (Burlington, MA, USA). Calibration curves were prepared in a concentration range of 5–500 mg L^-1^, using the wavelength of maximum absorption of each phenolic compound. The calibration curve of hydroxytyrosol was used for the quantification of DHPG, tyrosol, hydroxytyrosol acetate and 3,4-DHPEA-EDA, while the calibration curve of oleuropein was used for the quantification of oleuropein aglycon. These last compounds were identified according to Kanakis et al. (2013) [67] and Tasioula-Margari and Tsabolatidou (2015) [68]. Data acquisition, analysis and peak integration were performed using Chromeleon software (version 7.1; Dionex, Sunnyvale, CA, USA).

### 3.9. Statistical Analysis

Statistically significant differences between means were determined by analysis of variance (one-way ANOVA) followed by Tukey’s honestly significant difference (HSD, 5% level) post hoc test. These analyses were performed using the JMP statistical software v. Pro 14 (SAS Institute Inc., Cary, NC, USA). To evaluate the relationship between olive fruit and oil phenolic compounds and fatty acids, an OPLS-DA was performed, using the SIMCA software v. 14.1 (Umetrics, Umea, Sweden). The MFA of the fruit and oil phenolic compound data was performed using XLSTAT (Addinsoft, Anglesey, UK).

## 4. Conclusions

Considering the results obtained in the present study, we believe that both LC and ZL sustainable soil management strategies appear to be promising practices to implement in olive orchards under rainfed conditions, as an alternative to conventional tillage practice, since both were able to provide an adequate balance between crop yield and olive oil quality, simultaneously preserving soil quality under a climate change scenario. However, the innovative strategy of combining zeolites with leguminous cover crops, first reported in the present study, appears to confer advantages in terms of fruit and oil nutritional value. Our study contributes to a better understanding of the influence of different soil management practices on olive fruit and oil composition and quality. Nevertheless, more research is required to investigate their influence on soil-plant interactions, which also affect olive fruit and oil composition.

## Figures and Tables

**Table 1:** Crop yield (kg fruit tree^−1^) and fruit biometric parameters: fruit FW (g), pulp FW (g), pit FW (g), longitudinal length (mm), equatorial length (mm), fruit moisture (%) and pulp/pit ratio, as a function of soil treatment and harvest year.

	Crop Yield	Fruit FW	Pulp FW	Pit FW	Pulp/Pit Ratio	Long. Lenght	Equat. Length	Fruit Moisture
2018								
T	19.9 ± 1.45 ^b^	3.09 ± 0.131 ^b^	2.36 ± 0.122 ^b^	0.730 ± 0.019	3.25 ± 0.162	21.0 ± 0.457 ^b^	15.6 ± 0.322	55.9 ± 0.548 ^b^
LC	26.7 ± 1.91 ^a^	3.45 ± 0.069 ^a^	2.68 ± 0.056 ^a^	0.771 ± 0.016	3.48 ± 0.047	22.4 ± 0.191 ^a^	16.1 ± 0.135	59.4 ± 0.386 ^a^
ZL	27.5 ± 2.10 ^a^	3.17 ± 0.088 ^ab^	2.37 ± 0.086 ^ab^	0.798 ± 0.048	3.15 ± 0.165	21.8 ± 0.285 ^ab^	15.5 ± 0.149	56.1 ± 0.757 ^b^
*p*-value	0.014	0.035	0.027	n.s.	n.s.	0.014	n.s.	*p* < 0.001
2019								
T	13.6 ± 0.874	4.12 ± 0.128 ^b^	3.29 ± 0.109 ^b^	0.834 ± 0.024 ^ab^	3.95 ± 0.085	23.7 ± 0.288 ^b^	16.9 ± 0.196 ^ab^	55.7 ± 0.539 ^b^
LC	17.4 ± 1.79	4.64 ± 0.117 ^a^	3.74 ± 0.090 ^a^	0.906 ± 0.028 ^a^	4.16 ± 0.055	24.8 ± 0.221 ^a^	17.5 ± 0.164 ^a^	57.2 ± 0.260 ^a^
ZL	17.9 ± 1.44	3.98 ± 0.098 ^b^	3.18 ± 0.082 ^b^	0.804 ± 0.020 ^b^	3.97 ± 0.072	23.0 ± 0.262 ^b^	16.8 ± 0.152 ^b^	55.0 ± 0.336 ^b^
*p*-value	0.083	*p* < 0.0001	*p* < 0.0001	0.013	n.s.	*p* < 0.0001	0.008	0.001

Values are means ± SEM. Significance by Tukey’s HSD test: *p* < 0.05. Means with different superscript letters represent significant differences between treatments. Non-significant differences between treatments are represented by n.s.

**Table 2:** Olive fruit metabolite concentration as a function of soil management treatment and harvest year. Total phenols (TP, mg GAE g^−1^ DW), *ortho*-diphenols (mg GAE g^−1^ DW), flavonoids (mg CE g^−1^ DW) and total antioxidant capacity (TAC, mmol TE g^−1^ DW).

	TP	*ortho*-Diphenols	Flavonoids	TAC
2018				
T	35.4 ± 1.58	39.5 ± 0.871 ^a^	52.4 ± 3.75 ^a^	30.5 ± 0.532 ^a^
LC	31.5 ± 3.73	33.4 ± 0.48 ^b^	38.3 ± 2.64 ^b^	28.3 ± 0.877 ^ab^
ZL	34.7 ± 2.64	33.2 ± 1.21 ^b^	28.7 ± 3.52 ^b^	26.9 ± 1.05 ^b^
*p*-value	n.s.	*p* < 0.0001	*p* < 0.0001	0.019
2019				
T	19.3 ± 0.449	36.7 ± 1.37	12.2 ± 1.48	17.7 ± 0.408
LC	20.9 ± 0.427	36.3 ± 1.11	15.0 ± 1.29	18.3 ± 0.389
ZL	21.7 ± 0.967	41.4 ± 3.02	14.8 ± 1.56	17.3 ± 1.14
*p*-value	n.s.	n.s.	n.s.	n.s.

Values are means ± SEM. Significance by Tukey’s HSD test: *p* < 0.05. Means with different superscript letters represent significant differences between treatments. Non-significant differences between treatments are represented by n.s.

**Table 3:** Olive oil metabolite concentration as a function of soil management treatment and harvest year. Total phenols (TP, mg GAE kg^−1^), *ortho*-diphenols (mg GAE kg^−1^), flavonoids (mg CE kg^−1^) and total antioxidant capacity (TAC, mmol TE kg^−1^).

	TP	*ortho*-Diphenols	Flavonoids	TAC
2018				
T	234.5 ± 4.04 ^a^	58.5 ± 2.02 ^a^	171.6 ± 22.3 ^a^	155.0 ± 3.79 ^a^
LC	155.1 ± 4.92 ^b^	33.6 ± 3.05 ^c^	63.6 ± 9.38 ^b^	118.5 ± 3.06 ^b^
ZL	154.0 ± 6.25 ^b^	50.0 ± 1.40 ^b^	113.7 ± 18.3 ^ab^	134.5 ± 6.16 ^b^
*p*-value	*p* < 0.0001	0.001	0.001	*p* < 0.0001
2019				
T	159.7 ± 6.14 ^b^	59.4 ± 2.55 ^c^	73.2 ± 3.91	200.5 ± 13.4 ^c^
LC	188.1 ± 4.05 ^a^	99.9 ± 2.84 ^a^	91.4 ± 8.79	363.6 ± 8.85 ^a^
ZL	173.4 ± 3.07 ^ab^	85.6 ± 1.81 ^b^	84.5 ± 11.7	314.6 ± 12.3 ^b^
*p*-value	0.001	*p* < 0.0001	n.s.	*p* < 0.0001

Values are means ± SEM. Significance by Tukey’s HSD test: *p* < 0.05. Means with different superscript letters represent significant differences between treatments. Non-significant differences between treatments are represented by n.s.

**Table 4:** Climate characteristics recorded during 2018 and 2019 at the weather station in Paradela, close to the experimental plot. Average annual temperature (Tmean, °C), maximum temperature (Tmax, °C), minimum temperature (Tmin, °C), average temperature (°C) from the blossoming to ripening period (May–October, Tmean (May–October)), cumulative annual precipitation (ΣPrecp., mm) and cumulative precipitation (mm) from the blossoming to ripening period (May–Oct., ΣPrecp. (May–October)).

	Tmean	Tmax	Tmin	Tmean _(May–October)_	∑Precp.	∑Precp. _(May–October)_
2018	13.2	39.1	−7.9	18.6	708.4	125.8
2019	13.0	34.3	−7.9	18.2	652.2	179.8

**Table 5:** Olive fruit polyphenolic composition as a function of soil management treatment and harvest year. Hydroxytyrosol (HyTyr), tyrosol (Tyr), caffeic acid (Caff), chlorogenic acid (Chlorog), verbascoside (Verb), oleuropein (Oleur), gallocatechin (GCT), epigallocatechin (EGCT), epicatechin (EC), quercetin-3,7-di-*O*-glucoside (Querc), luteolin-3,7-di-*O*-glucoside (Lut-3,7-di-*O*-gluc), rutin, apigenin-7-*O*-glucoside (Api-7-*O*-gluc), apigenin (Api) and total phenols (TP) (mg kg^−1^ DW).

	Non-Flavonoid Composition						Flavonoid Composition								TP
	HyTyr	Tyr	Caff	Chlorog	Verb	Oleur	GCT	EGCT	EC	Querc	Lut-3,7-di-*O*-gluc	Rutin	Api-7-*O*-gluc	Api	
2018															
T	619.3 ± 2.99 ^a^	38.2 ± 0.659 ^a^	62.7 ± 0.305 ^b^	203.6 ± 2.16 ^a^	447.4 ± 5.90 ^b^	3940.5 ± 31.2 ^a^	208.0 ± 2.63 ^b^	101.4± 1.20 ^c^	155.2 ± 1.97 ^a^	3.34 ± 0.132 ^a^	1258.4 ± 8.32 ^a^	969.7 ± 7.71 ^a^	4.35 ± 0.161 ^b^	1.49 ± 0.060 ^a^	8013.6 ± 52.5 ^a^
LC	505.1 ± 5.62 ^b^	36.0 ± 1.09 ^a^	62.1 ± 0.632 ^b^	135.4 ± 1.14 ^c^	342.3 ± 3.99 ^c^	2751.7 ± 79.6 ^b^	365.2 ± 0.410 ^a^	159.2 ± 3.66 ^b^	101.1 ± 0.638 ^b^	2.09 ± 0.027 ^b^	862.2 ± 19.4 ^b^	545.7 ± 2.85 ^b^	6.50 ± 0.043 ^a^	0.212 ± 0.006 ^b^	5874.8 ± 103.1 ^c^
ZL	452.0 ± 7.44 ^c^	26.7 ± 0.603 ^b^	68.3 ± 0.669 ^a^	164.9 ± 0.325 ^b^	545.2 ± 2.78 ^a^	4164.8 ± 27.4 ^a^	200.3 ± 0.518 ^c^	188.5 ± 1.15 ^a^	157.6 ± 2.16 ^a^	1.44 ± 0.078 ^c^	748.8 ± 14.6 ^c^	466.4 ± 9.81 ^c^	4.24 ± 0.068 ^b^	0.206 ± 0.002 ^b^	7189.5 ± 8.73 ^b^
*p*-value	*p* < 0.001	*p* < 0.001	*p* < 0.001	*p* < 0.001	*p* < 0.001	*p* < 0.001	*p* < 0.001	*p* < 0.001	*p* < 0.001	*p* < 0.001	*p* < 0.001	*p* < 0.001	*p* < 0.001	*p* < 0.001	*p* < 0.001
2019															
T	303.4 ± 1.83 ^c^	12.4 ± 0.058 ^b^	64.4 ± 0.154 ^ab^	173.8 ± 1.74	153.7 ± 1.59 ^b^	1224.7 ± 5.35 ^b^	27.4 ± 0.289 ^c^	217.2 ± 3.28 ^a^	148.1 ± 0.607 ^b^	0.937 ± 0.001 ^c^	1048.1 ± 16.7 ^c^	525.1 ± 4.91 ^b^	2.48 ± 0.138 ^c^	0.199 ± 0.004 ^b^	3901.9 ± 22.5 ^b^
LC	427.1 ± 2.75 ^b^	4.47 ± 0.101 ^c^	63.6 ± 0.626 ^b^	167.9 ± 1.65	136.2 ± 2.19 ^b^	449.7 ± 8.37 ^b^	128.7 ± 1.73 ^a^	129.5 ± 0.205 ^b^	118.3 ± 1.64 ^c^	1.47 ± 0.018 ^b^	1257.8 ± 5.25 ^a^	385.9 ± 2.25 ^c^	3.39 ± 0.153 ^b^	1.47 ± 0.014 ^a^	3275.6 ± 7.22 ^b^
ZL	478.9 ± 5.06 ^a^	30.0 ± 1.15 ^a^	66.0 ± 0.260 ^a^	174.3 ± 1.19	225.9 ± 2.16 ^a^	2991.4 ± 329.8 ^a^	52.5 ± 2.74 ^b^	124.3 ± 1.33 ^b^	165.9 ± 0.635 ^a^	3.50 ± 0.043 ^a^	1136.5 ± 1.13 ^b^	662.2 ± 21.3 ^a^	4.59 ± 0.135 ^a^	0.225 ± 0.001 ^b^	6116.5 ± 340.5 ^a^
*p*-value	*p* < 0.001	*p* < 0.001	0.012	n.s.	*p* < 0.001	*p* < 0.001	*p* < 0.001	*p* < 0.001	*p* < 0.001	*p* < 0.001	*p* < 0.001	*p* < 0.001	*p* < 0.001	*p* < 0.001	*p* < 0.001

Values are means ± SEM. Significance by Tukey’s HSD test: *p* < 0.05. Means with different superscript letters represent significant differences between treatments. Non-significant differences between treatments are represented by n.s.

**Table 6:** Olive oil polyphenolic composition as a function of soil management treatment and harvest year. 3,4-Dihydroxyphenylglycol (DHPG); hydroxytyrosol (HyTyr); tyrosol (Tyr); hydroxytyrosol acetate (HyTyr acet.); oleuropein aglycone (Oleur. aglyc.); caffeic acid (Caff.); 3,4-DHPEA-EDA (dialdehydic forms of decarboxymethyl elenolic acid linked to hydroxytyrosol); verbascoside (Verb.); oleuropein (Oleur.); luteolin-7-*O*-glucoside (Lut.-7-*O*-gluc.); rutin; apigenin-7-*O*-glucoside (Api.-7-*O*-gluc.); luteolin (Lut.), apigenin (Api.) and total phenols (TP) (mg kg^−1^ DW).

	Non-Flavonoid Composition									Flavonoid Composition					TP
	DHPG	HyTyr	Tyr	HyTyr acet.	Oleur. aglyc.	Caff.	3,4-DHPEA-EDA	Verb.	Oleur.	Lut-7-*O*-gluc.	Rutin	Api-7-*O*-gluc.	Lut.	Api.	
2018															
T	0.159 ± 0.009 ^a^	0.065 ± 0.008 ^a^	0.171 ± 0.001 ^b^	0.607 ± 0.035 ^a^	14.3 ± 0.352 ^a^	0.035 ± 0.002 ^b^	9.70 ± 0.269 ^a^	0.855 ± 0.014 ^c^	1.29 ± 0.027 ^a^	0.262 ± 0.002	1.36 ± 0.004 ^b^	0.189 ± 0.006 ^c^	4.45 ± 0.222 ^a^	9.07 ± 0.058 ^a^	46.2 ± 0.650 ^a^
LC	0.115 ± 0.0002 ^b^	0.033 ± 0.002 ^b^	0.185 ± 0.003 ^b^	0.568 ± 0.001 ^ab^	10.8 ± 0.109 ^b^	0.027 ± 0.001 ^b^	6.57 ± 0.243 ^b^	1.37 ± 0.041 ^b^	1.04 ± 0.021 ^b^	0.246 ± 0.019	1.35 ± 0.009 ^b^	0.503 ± 0.040 ^a^	2.72 ± 0.079 ^b^	4.37 ± 0.083 ^b^	30.2 ± 0.161 ^c^
ZL	0.151 ± 0.010 ^a^	0.026 ± 0.003 ^b^	0.550 ± 0.014 ^a^	0.475 ± 0.023 ^b^	15.6 ± 0.707 ^a^	0.051 ± 0.003 ^a^	3.71 ± 0.215 ^c^	1.52 ± 0.002 ^a^	0.656 ± 0.020 ^c^	0.282 ± 0.005	1.49 ± 0.042 ^a^	0.363 ± 0.013 ^b^	4.66 ± 0.379 ^a^	3.98 ± 0.019 ^c^	33.5 ± 0.899 ^b^
*p*-value	0.017	0.003	*p* < 0.001	0.021	*p* < 0.001	*p* < 0.001	*p* < 0.001	*p* < 0.001	*p* < 0.001	n.s.	0.015	*p* < 0.007	0.003	*p* < 0.001	*p* < 0.001
2019															
T	0.201 ± 0.006 ^b^	0.003 ± 0.0004 ^c^	1.09 ± 0.011 ^c^	0.280 ± 0.007 ^b^	8.56 ± 0.168	0.009 ± 0.0003 ^b^	0.368 ± 0.009 ^a^	0.425 ± 0.015 ^b^	0.282 ± 0.0001 ^b^	0.261 ± 0.003 ^c^	1.34 ± 0.004	0.451 ± 0.001 ^b^	3.09 ± 0.442	2.75 ± 0.574	21.7 ± 0.626 ^b^
LC	0.215 ± 0.003 ^b^	0.030 ± 0.0004 ^a^	2.09 ± 0.024 ^b^	0.279 ± 0.013 ^b^	8.50 ± 0.142	0.017 ± 0.0004 ^a^	0.278 ± 0.0008 ^b^	0.664 ± 0.017 ^a^	0.317 ± 0.020 ^b^	0.387 ± 0.011 ^a^	1.34 ± 0.009	0.661 ± 0.006 ^a^	4.13 ± 0.494	4.82 ± 1.33	29.5 ± 1.61 ^a^
ZL	0.267 ± 0.012 ^a^	0.014 ± 0.0003 ^b^	2.52 ± 0.149 ^a^	0.346 ± 0.017 ^a^	8.29 ± 0.556	0.018 ± 0.0005 ^a^	0.281 ± 0.013 ^b^	0.706 ± 0.033 ^a^	0.516 ± 0.035 ^a^	0.323 ± 0.006 ^b^	1.33 ± 0.018	0.719 ± 0.046 ^a^	2.98 ± 0.667	4.66 ± 1.39	27.1 ± 2.22 ^ab^
*p*-value	0.003	*p* < 0.001	*p* < 0.001	0.017	n.s.	*p* < 0.001	*p* < 0.001	*p* < 0.001	*p* < 0.001	*p* < 0.001	n.s.	*p* < 0.001	n.s.	n.s.	0.038

Values are means ± SEM. Significance by Tukey’s HSD test: *p* < 0.05. Means with different superscript letters represent significant differences between treatments. Non-significant differences between treatments are represented by n.s.

**Table 7:** Olive fruit fat content (% DW) and fatty acid profile (%) as a function of soil management treatment and harvest year. Fat content, palmitic acid (C16:0), palmitoleic acid (C16:1), oleic acid (C16:1), linoleic acid (C18:2), linolenic acid (C18:3), oleic/linoleic acid ratio, saturated fatty acids (SFAs), unsaturated fatty acids (UFAs), monounsaturated fatty acids (MUFAs), polyunsaturated fatty acids (PUFAs) and UFA/SFA ratio.

	Fat Content	Palmitic Acid	Palmitoleic Acid	Oleic Acid	Linoleic Acid	Linolenic Acid	Oleic/Linoleic	SFA	MUFA	PUFA	UFA/SFA
2018											
T	50.1 ± 0.621 ^b^	14.3 ± 0.139	1.06 ± 0.078 ^a^	74.5 ± 0.308 ^b^	7.33 ± 0.226 ^a^	0.735 ± 0.106	10.2 ± 0.330 ^b^	15.9 ± 0.279	75.9 ± 0.375 ^b^	8.06 ± 0.211 ^a^	5.26 ± 0.108
LC	53.2 ± 0.106 ^a^	13.6 ± 0.123	0.819 ± 0.053 ^b^	76.4 ± 0.243 ^a^	6.36 ± 0.132 ^ab^	0.949 ± 0.153	12.0 ± 0.212 ^ab^	15.3 ± 0.177	77.4 ± 0.169 ^a^	7.31 ± 0.020 ^ab^	5.54 ± 0.073
ZL	53.8 ± 0.220 ^a^	14.1 ± 0.317	1.02 ± 0.008 ^ab^	76.3 ± 0.289 ^a^	6.02 ± 0.354 ^b^	0.483 ± 0.015	12.8 ± 0.829 ^a^	16.2 ± 0.323	77.5 ± 0.284 ^a^	6.51 ± 0.342 ^b^	5.20 ± 0.124
*p*-value	0.002	n.s.	0.041	0.006	0.027	n.s.	0.035	n.s.	0.014	0.009	n.s.
2019											
T	60.6 ± 0.364 ^a^	12.7 ± 0.068 ^b^	0.773 ± 0.051	75.1 ± 0.615	8.38 ± 0.241 ^a^	0.593 ± 0.028	8.98 ± 0.333 ^b^	15.0 ± 0.290 ^b^	76.0 ± 0.555	8.97 ± 0.266 ^a^	5.67 ± 0.129 ^a^
LC	52.6 ± 0.289 ^b^	13.1 ± 0.221 ^ab^	0.871 ± 0.042	77.4 ± 2.05	7.76 ± 0.212 ^a^	0.641 ± 0.059	9.98 ± 0.356 ^b^	15.5 ± 0.335 ^ab^	78.4 ± 2.05	8.18 ± 0.142 ^a^	5.43 ± 0.126 ^ab^
ZL	54.2 ± 1.82 ^b^	14.7 ± 0.749 ^a^	0.885 ± 0.017	76.1 ± 0.629	5.00 ± 0.094 ^b^	0.559 ± 0.053	15.2 ± 0.163 ^a^	17.3 ± 0.781 ^a^	77.1 ± 0.667	5.56 ± 0.115 ^b^	4.79 ± 0.252 ^b^
*p*-value	0.005	0.045	n.s.	n.s.	*p* < 0.001	n.s.	*p* < 0.001	0.044	n.s.	*p* < 0.001	0.033

Values are means ± SEM. Significance by Tukey’s HSD test: *p* < 0.05. Means with different superscript letters represent significant differences between treatments. Non-significant differences between treatments are represented by n.s.

**Table 8:** Olive oil quality indices as a function of soil management treatments and harvest year. Free acidity (FA, %), peroxide index (PI, mEq of O_2_ kg^−1^), K232, K270 and ΔK.

	FA	PI	K232	K270	∆K
2018					
T	0.281 ± 0.012 ^a^	17.1 ± 1.12 ^a^	2.49 ± 0.089 ^a^	0.218 ± 0.003 ^a^	0.005 ± 0.003 ^a^
LC	0.229 ± 0.007 ^b^	12.3 ± 0.733 ^b^	2.15 ± 0.173 ^a^	0.171 ± 0.002 ^b^	0.004 ± 0.0001 ^b^
ZL	0.241 ± 0.003 ^b^	7.99 ± 0.823 ^c^	1.62 ± 0.031 ^b^	0.178 ± 0.002 ^b^	0.003 ± 0.00001 ^b^
*p*-value	0.009	0.001	0.005	*p* < 0.001	0.004
2019					
T	0.080 ± 0.0001 ^b^	3.79 ± 0.683	2.63 ± 0.034	0.171 ± 0.017	0.007 ± 0.002
LC	0.147 ± 0.013 ^a^	2.36 ± 0.126	2.77 ± 0.069	0.185 ± 0.007	0.005 ± 0.00001
ZL	0.120 ± 0.0002 ^a^	2.78 ± 0.049	2.65 ± 0.032	0.177 ± 0.003	0.005 ± 0.0001
*p*-value	0.002	n.s.	n.s.	n.s.	n.s.

Values are means ± SEM. Significance by Tukey’s HSD test: *p* < 0.05. Means with different superscript letters represent significant differences between treatments. Non-significant differences between treatments are represented by n.s.

**Table 9:** Selected properties of zeolites used in this experiment (data provided by the manufacturer).

Mineral Properties		Physicochemical Properties			
Clinoptilolite	88.0–95.0%	SiO_2_	65.0–71.3%	CEC	1.5–1.9 mEq/g
Feldspar	3.0–5.0%	Al_2_O_3_	10.0–12.0%	Porosity	45.0–50.0%
Montmorillonite	2.0–5.0%	CaO	2.5–3.7%	Specific surface	70.0–80.0 m^2^g^−1^
Cristobalite	0–2.0%	K_2_O	2.3–3.5%	pH	7.0–8.0
Volume density	2000–2400 Kg/m^3^	FeO_3_	0.8–1.9%	Thermic stability	>450 °C
MOHS hardness	2.0–3.0	MgO	0.9–1.2%	Chemical stability	3.0 < pH < 11.0
Granulometry	0.6–1.5 mm	Na_2_O	0.3–0.65%	Apparent density	0.85 g cm^−3^
		TiO_2_	0–0.10%		

## Data Availability

The data presented in this study are available on request from the corresponding author.

## Supplementary Materials

The following supporting information can be downloaded at: https://www.mdpi.com/article/10.3390/molecules28062545/s1, Table S1: CV-ANOVA respective to the OPLS-DA of olive fruit phenolic compounds; Figure S1: Permutation tests of OPLS-DA relative to the olive fruit phenolic compounds according to (**a**) T, (**b**) LC and (**c**) ZL treatments; Table S2: CV-ANOVA respective to the OPLS-DA of olive oil phenolic compounds; Figure S2: Permutation tests of OPLS-DA relative to the olive fruit phenolic compounds, according to (**a**) T, (**b**) LC and (**c**) ZL treatments; Figure S3: Correlation between the olive fruit total phenolic compounds in relation to olive oil phenolic compounds; Table S3: CV-ANOVA respective to the OPLS-DA of olive fruit fatty acids; Figure S4: Permutation tests of OPLS-DA relative to the olive fruit fatty acids, according to (**a**) T, (**b**) LC and (**c**) ZL treatments.
