# Supplementary material for: Combining Zeolites with Early-Maturing Annual Legume Cover Crops in Rainfed Orchards: Effects on Yield, Fatty Acid Composition and Polyphenolic Profile of Olives and Olive Oil
Source: Molecules. 2023 Mar 10;28(6):2545. doi: 10.3390/molecules28062545 (PMC10054706; doi:10.3390/molecules28062545)
Supplement: Supplementary file 1 [file molecules-28-02545-s001.zip › molecules-2203001-supplementary.pdf]

Combining Zeolites with Early-Maturing Annual Legume Cover Crops in Rainfed Orchards: Effects on Yield, Fatty Acid Composition and Polyphenolic Profile of Olives and Olive Oil

Sandra Martins [1], Ermelinda Silva [1,2], Cátia Brito [1,3], Luís Pinto [2], Carlos Martins-Gomes [1,4], Alexandre Gonçalves [2], Margarida Arrobas [5], Manuel Ângelo Rodrigues [5], Carlos M. Correia [1,3] and Fernando M. Nunes [4,*]

1 CITAB—Centre for the Research and Technology of Agro-Environmental and Biological Sciences, University of Trás-os-Montes and Alto Douro, 5000-801 Vila Real, Portugal
2 MORE—Collaborative Laboratory Mountains of Research, Brigantia Ecopark, 5300-358 Bragança, Portugal
3 Inov4Agro—Institute for Innovation, Capacity Building and Sustainability of Agri-Food Production, University of Trás-os-Montes and Alto Douro, 5000-801 Vila Real, Portugal
4 CQ-VR—Food and Wine Chemistry Laboratory, Chemistry Research Centre–Vila Real, University of Trás-os-Montes e Alto Douro, 5000-801 Vila Real, Portugal
5 CIMO—Centro de Investigação de Montanha, Instituto Politécnico de Bragança, 5300-253 Bragança, Portugal
* Correspondence: fnunes@utad.pt

Supplementary Results

Table S1. CV-ANOVA respective to the OPLS-DA of olive fruit phenolic compounds.

M1(Untitled)	SS	DF	MS	F	p	SD
Total Corr.	34	34	1			1
Regression	33.5831	18	1.86573	71.5989	6,02805e-012	1.36592
Residual	0.416928	16	0.026058			0.161425

a)

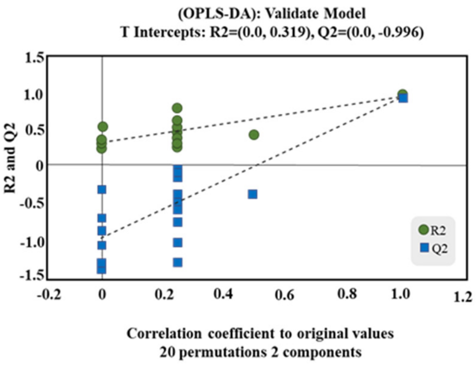

b)

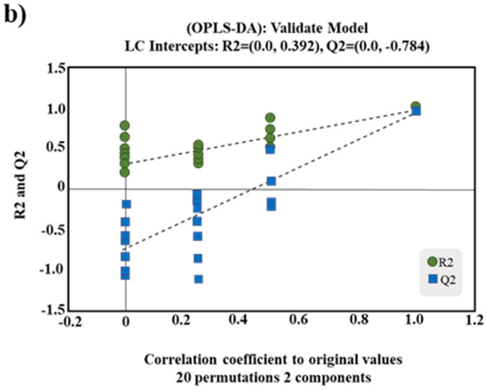

c)

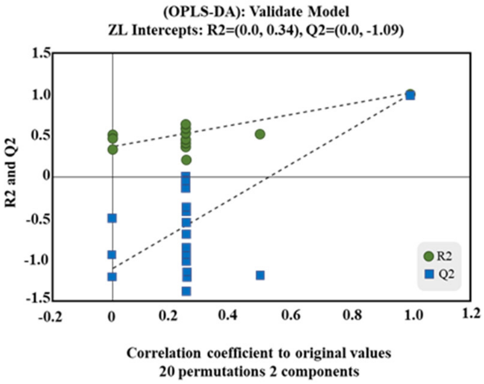

Figure S1. Permutation tests of OPLS-DA relative to the olive fruit phenolic compounds according to T **(a)**, LC **(b)** and ZL **(c)** treatments.

Table S2. CV-ANOVA respective to the OPLS-DA of olive oil phenolic compounds.

M1(Untitled)	SS	DF	MS	F	p	SD
Total Corr.	34	34	1			1
Regression	27.6474	18	1.53597	3.86857	0.00453686	1.23934
Residual	6.3526	16	0.397038			0.630109

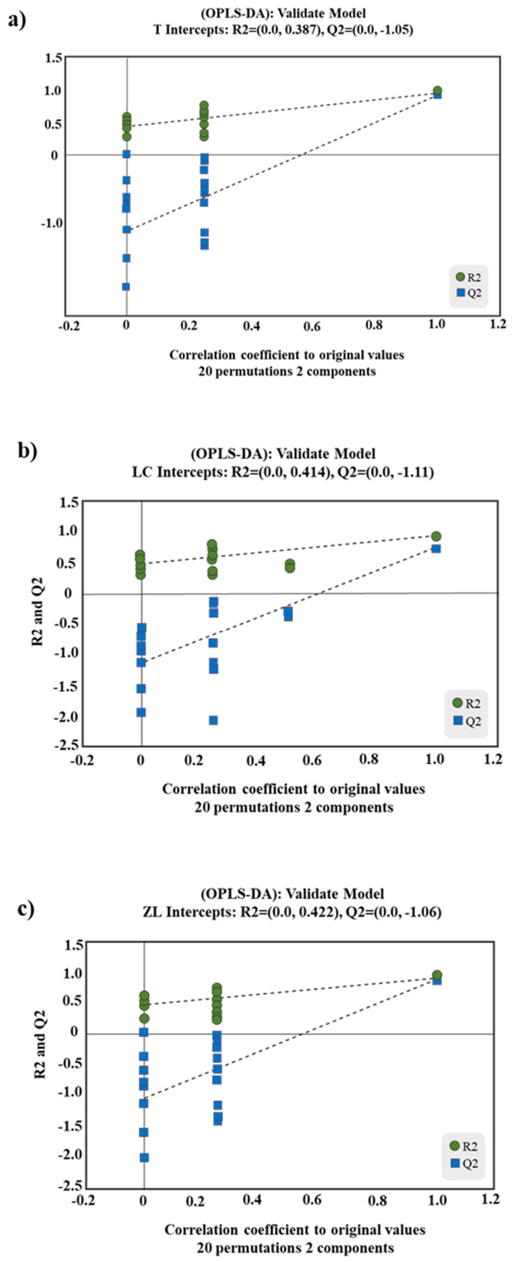

Figure S2. Permutation tests of OPLS-DA relative to the olive oil phenolic compounds, according to T **(a)**, LC **(b)** and ZL **(c)** treatments.

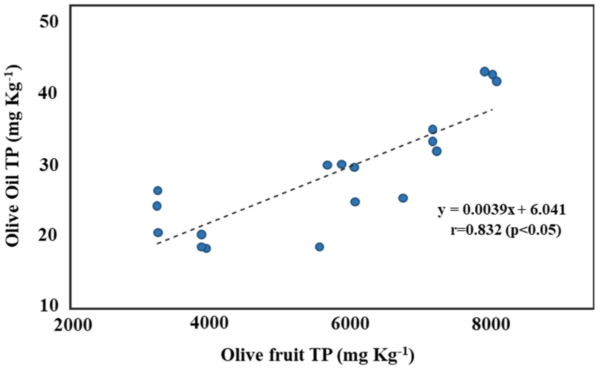

Figure S3. Correlation between the olive fruit total phenolic compounds in relation to olive oil phenolic compounds.

Table S3. CV-ANOVA respective to the OPLS-DA of olive fruit fatty acids.

M1(Untitled)	SS	DF	MS	F	p	SD
Total Corr.	34	34	1			1
Regression	12.1603	8	1.52003	1.80959	0.120777	1.2329
Residual	21.8397	26	0.83999			0.91651

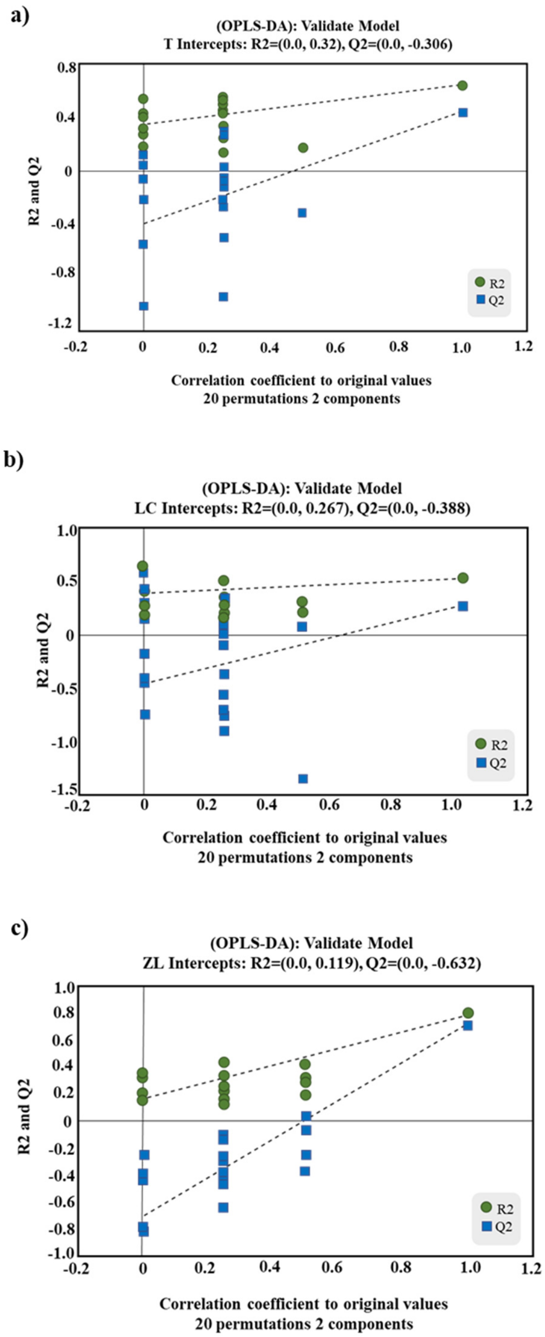

Figure S4. Permutation tests of OPLS-DA relative to the olive fruit fatty acids, according to T **(a)**, LC **(b)** and ZL **(c)** treatments.
